# Comment on Warrick, B.J., Boyer, L.V., Seifert, S.A. Non-Native (Exotic) Snake Envenomations in the U.S., 2005–2011. *Toxins* 2014, *6*, 2899–2911

**DOI:** 10.3390/toxins6123258

**Published:** 2014-12-05

**Authors:** Jiri Valenta, Pavel Michalek, Zdenek Stach

**Affiliations:** Department of Anesthesiology and Intensive Medicine, 1st Medical Faculty of the Charles University and General University Hospital, U nemocnice 2, 128 08 Prague, Czech Republic; E-Mails: pavel.michalek@vfn.cz (P.M.); zdenek.stach@vfn.cz (Z.S.)

We have read with interest the article by Warrick *et al.*, which was recently published in this journal [1]. The authors provided in their discussion reference and some comments to our article published in the journal “Clinical Toxicology” earlier this year [2]. We appreciate their interest in our work, however, we would like to correct some information provided by Warrick *et al*.

The authors cite that we treated 34 non-native envenomations in the surveyed period. However, we would like to correct that, in reality, 72 envenomations (43 local envenomations and 29 systemic envenomations) were treated in our institution over the 15-year period. Warrick also stated that 31 different venomous snakes caused envenomations in our cohort. The envenomations were actually caused by 34 different venomous snakes, including 31 reliably identified species and three identified genera (two patients were bitten by *Naja* species, while one patient was envenomed by *Atheris* species).

Warrick *et al.* also stated that “in at least one report, it took five days to obtain an antivenom”. In this particular case, the male patient attended a local hospital as late as five days after snakebite caused by Mangshan pitviper (*Protobothrops mangshanensis*) because of development of a large hematoma on his upper extremity, contralateral to the site of the original snakebite. A paraspecific antivenom (specific antivenom does not exist) was immediately available and this was administered the same day. 

We believe that this corrected data may be beneficial for the readers of *Toxins*.
